# Folic Acid and Autism: A Systematic Review of the Current State of Knowledge

**DOI:** 10.3390/cells10081976

**Published:** 2021-08-03

**Authors:** Bianka Hoxha, Malvina Hoxha, Elisa Domi, Jacopo Gervasoni, Silvia Persichilli, Visar Malaj, Bruno Zappacosta

**Affiliations:** 1 Department of Chemical-Pharmaceutical and Biomolecular Technologies, Faculty of Pharmacy, Catholic University “Our Lady of Good Counsel”, Rruga Dritan Hoxha, 1000 Tirana, Albania; b.hoxha@unizkm.al; 2 Department for Chemical-Toxicological and Pharmacological Evaluation of Drugs, Faculty of Pharmacy, Catholic University “Our Lady of Good Counsel”, Rruga Dritan Hoxha, 1000 Tirana, Albania; e.domi@unizkm.al (E.D.); b.zappacosta@unizkm.al (B.Z.); 3 Area Diagnostica di Laboratorio UOC Chimica, Biochimica e Biologia Molecolare Clinica Fondazione Policlinico Universitario A. Gemelli IRCCS, Largo A. Gemelli 8, 00168 Rome, Italy; jacopo.gervasoni@unicatt.it (J.G.); silvia.persichilli@unicatt.it (S.P.); 4 Department of Economics, Faculty of Economy, University of Tirana, 1000 Tirana, Albania; visarmlaj@feut.edu.al

**Keywords:** folic acid, autism, methylene tetrahydrofolate reductase, autism spectrum disorders

## Abstract

Folic acid has been identified to be integral in rapid tissue growth and cell division during fetal development. Different studies indicate folic acid’s importance in improving childhood behavioral outcomes and underline its role as a modifiable risk factor for autism spectrum disorders. The aim of this systematic review is to both elucidate the potential role of folic acid in autism spectrum disorders and to investigate the mechanisms involved. Studies have pointed out a potential beneficial effect of prenatal folic acid maternal supplementation (600 µg) on the risk of autism spectrum disorder onset, but opposite results have been reported as well. Folic acid and/or folinic acid supplementation in autism spectrum disorder diagnosed children has led to improvements, both in some neurologic and behavioral symptoms and in the concentration of one-carbon metabolites. Several authors report an increased frequency of serum auto-antibodies against folate receptor alpha (FRAA) in autism spectrum disorder children. Furthermore, methylene tetrahydrofolate reductase (MTHFR) polymorphisms showed a significant influence on ASD risk. More clinical trials, with a clear study design, with larger sample sizes and longer observation periods are necessary to be carried out to better evaluate the potential protective role of folic acid in autism spectrum disorder risk.

## 1. Introduction

Autism spectrum disorders (ASDs) are complex neurodevelopmental disorders, characterized by social and communication impairments, sensory hyper-sensitivity, and difficulties adjusting to unexpected change, as well as restricted interests and repetitive behaviors. ASDs are estimated to affect up to 3% of children in the United States with an overall prevalence of 13.4 per 1000 children aged 4 years in 2010, 15.3 in 2012, and 17.0 in 2014 [1].

Many etiologic and risk factors, including genetic and environmental influences, have been proposed over time for autism and ASDs. Genetic studies have focused, for several years, on single gene mutations or small groups of genes but, despite about 600 genes considered, only a small number of genes, such as Fragile X, SHANK3, and CASPR2, have shown a relevant association with ASDs. More recently, ongoing studies are investigating the role of different genetic mechanisms and on the whole exome sequencing [2], and overall, the multifactorial theory of ASDs risk has been broadly accepted, as well of other neuropsychiatric diseases, providing for the involvement of both hundreds of genes and environmental factors [3] in subjects showing a genetic predisposition [4].

The most frequently considered environmental risk factors for ASDs that can influence the epigenome include preconception and prenatal maternal lifestyle, maternal metabolism, nutrition [5], medication, toxic and pollutants exposure [6], socio-economic conditions, pregnancy and delivery complications, and perinatal events, such as birth complications [7].

A growing interest is also surrounding the environmental risk factors–epigenetic mechanisms correlation in the pathogenesis of ASDs; it is possible, in fact, that ASD risk enhances due to a synergistic effect of inherited genes in combination with environmental exposure; epigenetic mechanisms, especially DNA methylation and histone modification, can influence gene expression without changing the DNA sequence and could be likely considered mediators in the onset of neurodevelopmental diseases [8].

The epigenetic dependent modifications in the brain, such as neuron connectivity and morphology, occur most frequently during pregnancy and in perinatal period [9].

In the last two decades, many studies have investigated the possible correlation between morphological and functional alterations of the brain and some clinical aspects of ASDs. Based on postmortem analyses, neuroimaging findings and animal models, these studies focused especially on cerebellum, frontal cortex, and amygdala, highlighting some anatomic alterations, such as prefrontal cortex overgrowth, multifocal cortical dysplasias, or functional anomalies regarding the synapse function and the local connectivity in different brain areas [10].

Many efforts should be made to identify possible markers of ASDs, and interesting findings are emerging from metabolic and biochemical studies. Several authors have considered the relationship between ASD clinical symptoms and gut microbiota, which could influence neurodevelopment if dysbiosis is present [11]. Moreover, the complex involvement of mitochondria in ATP production and neural function has been also investigated, and their dysfunction has been associated with the neurodevelopmental clinical aspects due to the respiratory electron transport chain defects [12] in well-defined brain areas such as the cerebellum, frontal cortex, and temporal cortex [13].

Some components of the one-carbon metabolism, such as folate, methionine, and choline, play an unquestionable role in DNA methylation as methyl donors [14]. However, the cooperation between genes and environmental factors can alter DNA and then enhance ASD risk also by inducing de novo mutational events or de novo replication defects. The epigenetic mechanisms might then represent a reliable link between environmental influence and neurodevelopmental diseases [15,16], but to consolidate this hypothesis, other large studies are probably necessary.

**Figure 1:**
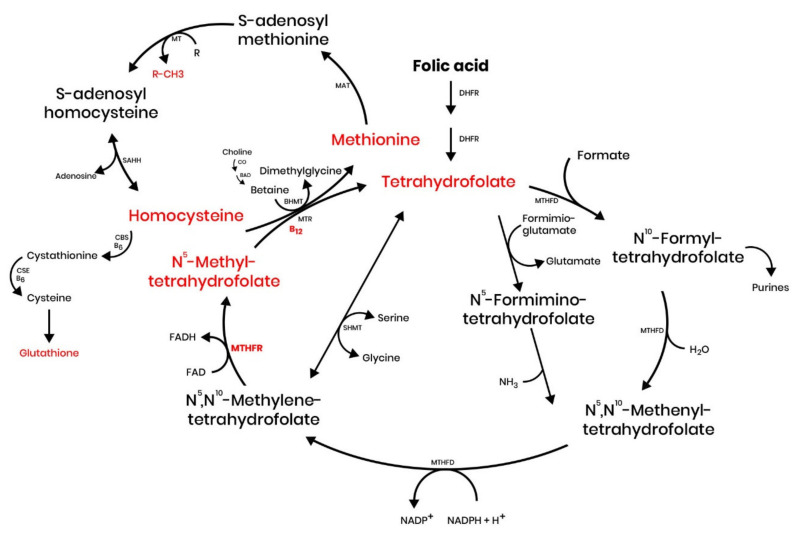
Overview of the one-carbon metabolism. Abbreviations: dihydrofolate reductase (DHFR); serine hydroxymethyltransferase (SHMT); methylenetetrahydrofolate reductase (MTHFR); multiple methyltransferases (MT); S-adenosylhomocysteine hydrolase (SAHH); cystathionine beta-synthase (CBS); methionine synthase (MTR); betaine aldehyde dehydrogenase (BAD); methionine adenosyltransferase (MAT); betaine homocysteine S-methyltransferase (BHMT); cystathionine γ-lyase (CSE); methylenetetrahydrofolate dehydrogenase (MTHFD).

Folate, a water-soluble B group vitamin naturally present in a wide variety of foods in different concentrations, is an essential nutrient that supports multiple physiological processes. Different forms of folate participate in different important reactions, such as DNA methylation and replication. As a cofactor in a multitude of single-carbon transfer reactions, it has a direct influence in biosynthesis, epigenetic maintenance, amino acid homeostasis, and redox defense [17,18]. FA, as a synthetic compound is metabolized differently to naturally occurring forms of folates with a bioavailability approximately 70% higher [19] (Figure 1). Indeed, reduced folate levels are linked to a variety of clinical conditions, such as neurological and cardiovascular diseases, and an increased risk of NTDs [20,21,22].

During neurogenesis and cell migration in most cortical and subcortical structures, high concentrations of methyl donors are required [23]. Several studies have been performed about the possible causative relationship between folate intake, pre or during pregnancy, its metabolism, and the onset of ASDs because of the modulation that cell folate exerts on the developing brain through the synthesis of DNA, neurotransmitters, and myelination. The results, however, are unfortunately still conflicting.

Two main MTHFR single nucleotide polymorphisms (SNPs), cytosine to thymidine switch at nucleotide position 677 and adenosine to cytosine switch at nucleotide position 1298, have been linked to functional alterations of folate levels [24]. The attenuation of this enzyme activity, prevalently due to the 677C → T mutation, leads to impaired methylation reactions and nucleotide synthesis. The abnormal one-carbon metabolism related to the MTHFR polymorphism might result in a hyperhomocysteinemia and hypomethylation function related to the increased synthesis of S-adenosyl homocysteine (AdoHcy) [25]. The influence of MTHFR genotype in serum folate and homocysteine levels has been associated to several neurologic and psychiatric traits [26]. Furthermore, the study of tissue concentrations of one-carbon metabolites in the liver, cerebral cortex, basal forebrain, and of MTHFR genotyping in mice shows that MTHFR deficiency could increase the risk of ASD-like behavior, underlining the relevance of the prenatal dietary intervention focused on MTHFR genotypes [27]. In addition, a diminished red blood cell (RBC) folate uptake and a decreased serum folate level resulting in an irregular one-carbon metabolism has been associated with the AG and GG genotypes of the reduced folate carrier (RFC1) [28,29].

Modulation of folate uptake at the blood–brain barrier (BBB) through RFC1, folate receptor alpha (FRα), or proton-coupled folate transporter (PCFT) may have clinical importance in establishing optimal therapies for childhood neurodegenerative disorders caused as a result of the inactivation or mutations of these folate transport systems [30].

Human and animal experimental studies have also been conducted to evaluate the role of auto-antibodies against FRα, blocking at the choroid plexus 5-methyltetrahydrofolate (5-MTHF) transfer to the brain, and their association with pregnancy-related complications, as well as neurodevelopmental disorders. Ramaekers V. et al., showed that reduced cerebrospinal fluid (CSF) folate could be explained by serum FR autoantibodies blocking the folate binding site of the membrane-attached FR on the choroid epithelial cells, while oral folinic acid supplements (starting dose of 1.0 mg/kg/day), as a 5-formyl derivative that does not require the action of dihydrofolate reductase for its conversion, can lead to normal CSF 5-MTHF and partial or complete clinical recovery after 12 months [31].

The aim of this systematic review is to evaluate the current state of knowledge on the potential role of FA in ASDs, both in animal and human studies.

## 2. Materials and Methods

This systematic review was conducted rigorously following the preferred reporting items for systematic reviews (PRISMA) guidelines [32]. We collected relevant data conformed to the eligibility criteria of our study.

### 2.1. Study Design

According to previous published evidence of FA deficiency involvement in ASDs events [33], we conducted a systematic review to assess the association of FA with ASDs in both animal and human clinical studies.

### 2.2. Eligibility Criteria 

Predefined eligibility criteria for inclusion of the studies were as follows: all published randomized controlled trials (RCTs), observational studies (cohort or case-control design), and reviews (Table 6) dealing with the association between FA and ASDs. The following events were considered as primary outcomes: maternal exposure to FA and/or multivitamin supplements and ASD risk in offspring; improvement of autism symptoms towards sociability, cognitive verbal/preverbal, receptive language, and affective expression and communication; improvement of verbal communication; development of language and communication skills; MTHFR genetic variants as a risk factor for ASDs; and the effect of exposure to FRα auto-antibodies. Secondary outcomes included: serum homocysteine levels; serum folate and vitamin B12 levels; glutathione metabolism before and after treatment; CSF and serum 5-MTHF levels; plasma levels of: methionine, S-adenosyl-methionine (SAM), S-adenosyl-homocysteine (SAH), SAM:SAH ratio, cysteine, and cysteinylglycine.

Our search without language limitation reviewed articles with no restriction by year of publication or age of the patients. All published data until 31 May 2021 were included. Abstracts, conference papers, posters, and in vitro studies have not been considered.

### 2.3. Literature Search and Selection of Articles 

**Figure 2:**
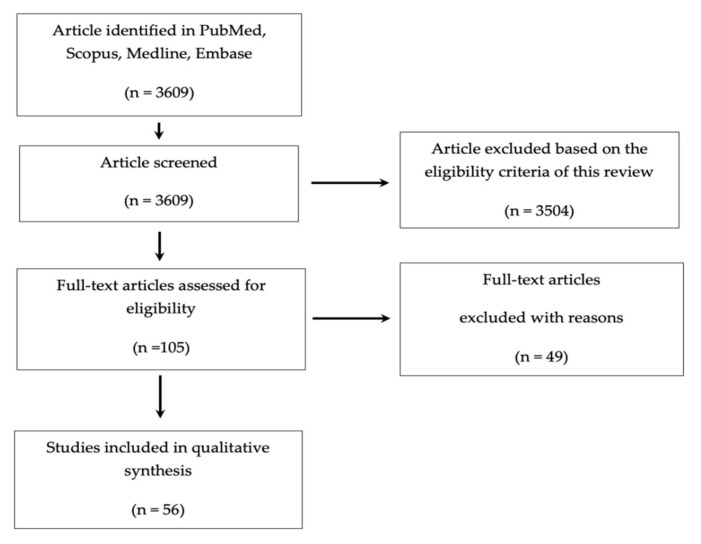
Prisma flow diagram: schematic diagram of literature search and selection for articles included in this systematic review.

We searched in PubMed, Scopus, Medline, and Embase databases using different key words to identify all studies that indicated the association between FA and ASDs. Specific text words were used: “folic acid and autism”, “folic acid” AND “autism”, “polymorphism and autism”, “MTHFR polymorphism and autism”, “antibodies FRA and autism”, “autism and folic acid absorption”, “autism and folic acid metabolism”, “cholecalciferol and folic acid”, “folic acid deficiency and autism”, “FRAs and autism”, “smoking and folic acid and autism”, “western people and autism”, “women in reproductive age and autism”, “folate and autism”, “folate” AND “autism”, “autism and folate absorption”, “autism and folate metabolism”, “cholecalciferol and folate”, “folate deficiency and autism”, “smoking and folate and autism”. After data extraction, we reviewed the titles and the respective abstracts for all records. Two authors independently reviewed the full texts to further assess if the selected studies fulfilled the eligibility criteria, verifying the results and removing duplicates. No disagreements were noted between the two authors. In the last phase, two authors independently evaluated the studies that met the criteria. Figure 2 reports a schematic diagram of the literature search procedure.

### 2.4. Data Extraction 

Fifty six articles met the criteria and were selected for inclusion in our systematic review. Data extracted from each eligible article included the study name, publication year, main outcome, study outcome parameters, sample size, type of study, age of children, effect of folic acid, supplementation used, period of folic acid intake, and follow up period. In regard to animal studies, the type of animal and treatment used were reported.

### 2.5. Risk of Bias across Studies

In the majority of studies reviewed, there was the possibility of bias due to the small sample sizes and different case-control study designs that could potentially limit the sensitivity of the analyses to spot the treatment effects. The lack of reliable biomarkers that could specifically estimate who may benefit from a certain intervention is another factor that is needed to better combine treatment protocols. In studies investigating the prenatal use of FA, different unmeasured factors that could impact the background of ASD risk in offspring were not estimated, such as medication use during pregnancy, parental age, parental education, maternal exercise, maternal body mass index, maternal toxicant exposures, whether the pregnancy was planned, maternal smoking during pregnancy, weight gain during pregnancy, and year of birth. A possible misclassification due to insufficient information on gestational age may actually decrease the accuracy of exposure classifications. Also, a misclassifications of FA exposure as a result of not recorded maternal supplementation use might confound the evaluation of the risk reduction. Another limitation was the unavailable information of mother’s whole blood and serum folate levels in several studies.

The phenotypic expression of MTHFR genotype might change during FA supplementation. In addition, in the genetic studies, there was a lack in the evaluation of serum folate levels or folate intake in cases and controls. Also, a further limitation was the absence of estimation in most studied cases and controls of vitamin B12, folate, and their metabolites serum levels.

In the animal studies, the limited sample sizes and the use of different mouse strains or pups from a single dam could possibly influence the tested outcomes.

## 3. Results

### 3.1. Overview of Literature Search Results

A total number of 3609 articles were identified with our search strategy as shown in Figure 2. We eliminated duplicates and other studies not eligible for different reasons, such as in vitro studies, reviews and/or articles not correlated with the main argument (FA and ASDs). Finally, we systematically reviewed 56 studies.

### 3.2. Summary of the Results Reported by Human Clinical Trials Included in the Systematic Review

Studies regarding the association between the use of FA and ASDs lacked homogeneity due to a number of factors, including FA intake (mainly assessed by telephone interviews and self-reported questionnaire/form), period of intake, variability in the measured outcome parameters, and the specification of FA supplement used.

The essential characteristics of all human clinical studies systematically reviewed are summarized in Table 1, Table 2, Table 3 and Table 4 according to specific primary outcome effects, such as the association between maternal FA supplementation and the risk of ASDs in offspring (10 studies); folate status or clinical benefits observed after folate supplementation in ASD diagnosed children (nine studies); role of MTHFR Gene C677T polymorphism in ASDs risk (10 studies); and frequency of serum FRAA in ASDs children (three studies).

### 3.3. Studies on the Association between Maternal FA Supplementation in Reducing the Risk of Asds in Offspring

In Table 1, the results of Roth et al. [43], in the Norwegian mother and child cohort study are reported: they observed a lower risk of language disability following preconception maternal FA supplementation in 3 year old children; in 2017, in the randomized controlled trial by Christian et al. [42] a positive effect between maternal FA supplementation and neurological aspects was found. The Stockholm study [37] indicates that maternal multivitamin supplementation during pregnancy may be associated to a reduced risk of ASDs with intellectual disability by 0.26% (158 cases out of 61,934), with respect to 0.48% (430 cases out of 90,480) in the no nutritional supplementation use group. Nilsen et al., further observed that maternal prenatal FA supplement use was associated with a 14–17% adjusted risk reduction for ASDs [41]. The FA supplement dose was not specified in all studies, and the intake period was also variable. No correlation between early folate or multivitamin intake for ASDs was found in respect to women who did not have a supplement use in the same period [34]. This study has several limitations, such as the inability to assess FA use in the absence of multivitamin or other supplement use reflecting insufficient folate intake to accomplish levels necessary to obtain the desired protective effect. In addition, it suggested no ASDs risk reduction in one population at the time period this study was conducted (folic acid-adjusted risk ratio: 1.06, 95% confidence interval: 0.82–1.36; multivitamin-adjusted risk ratio: 1.00, 95% confidence interval: 0.82–1.22) [34].

In a case-control cohort study of 45,300 Israeli children, with 572 (1.3%) ASDs diagnosed cases, Levine et al., observed a statistically significant association between maternal FA or multivitamin supplement use before pregnancy (RR, 0.39; 95% CI, 0.30–0.50; *p* < 0.001) and/or during pregnancy (RR, 0.27; 95% CI, 0.22–0.33; *p* < 0.001) and reduced risk of ASDs in offspring [35]. In a group of 85,176 children from the Norwegian mother and child cohort study (MoBa) [36], Surén et al., found that the use of prenatal FA supplements around the time of conception was associated with an approximately 45% lower risk of autistic disorder. In a recent paper, Raghavan et al., analyzed the maternal plasma levels of folate, vitamin B12, and homocysteine from samples taken at birth in a cohort study of 1257 mother–child pairs [38]. Furthermore, the authors correlated the risk of ASDs with the frequency of prenatal multivitamin supplementation (low: ≤2 times/week; high: >5 times/week). Interestingly, a “U” shaped relationship was found between the frequency of maternal supplementation and risk of ASDs: this was reduced following moderate intake (3–5 times/week), while increased in case of low or high frequency. Likewise, the risk of ASDs was higher in children from mothers whose plasma levels of folate and B12 were elevated (>90th percentile) [38]. 

However, another study found that prenatal FA use was associated with less child autistic traits, while no association of autistic traits in the offspring was found with maternal plasma folate concentration measured in early pregnancy [40]. The risk of ASDs in children whose mothers had, at delivery, very high concentrations of plasma folate and B12 (≥90th percentile) could find an explanation in the sensitivity of the fetus brain exposed to higher levels of micronutrients, especially in the third trimester when some relevant neurological processes are ongoing.

In the recent MARBLES study (markers of autism risk in babies: learning early signs), Schmidt analyzed data from children (n = 332) and their mothers (n = 305) to evaluate the association between risk of ASDs recurrence and maternal prenatal multivitamin use [39]. Women that took prenatal FA (~600 μg) in the first month of pregnancy had children that were half as likely to receive an ASDs diagnosis (adjusted RR, 0.50; 95% CI, 0.30–0.81), with significant lower autism symptom severity (adjusted estimated difference, −0.60; 95% CI, −0.97 to −0.23) compared to children whose mothers did not take prenatal vitamins in the first month.

### 3.4. Studies of Folate Supplementation or Folate Levels in ASD Diagnosed Children

In a recent randomized controlled single-blind study of 67 children and adults with ASDs from Arizona vs. 50 non-sibling neurotypical controls of similar age and gender, it was confirmed a significant improvement in nonverbal intellectual ability in the treatment group (~600 μg FA) compared to the non-treatment group by using different tests, such as IQ (+6.7 ± 11 IQ points vs. −0.6 ± 11 IQ points, *p* = 0.009) and non-verbal intelligence index (+10% in treatment group vs.−1%, *p* value 0.01) based on a blinded clinical assessment (Table 2) [49]. The treatment group had significantly higher improvement in autism symptoms and developmental age with an increased level of docosahexaenoic acid (DHA); eicosapentaenoic acid (EPA); carnitine; Coenzyme Q10; and vitamins A, B2, B5, B6, B12, FA, observed as compared to the non-treatment group. Nutritional status, non-verbal IQ, autism symptoms, and other symptoms in most ASDs patients were improved, based on the semi-blinded assessment, suggesting the efficacy of a comprehensive nutritional and dietary intervention.

Improvement in verbal communication, motor skills, and plasma levels of homocysteine, FA, B12, glutathione following FA (400–600 μg) or folinic acid (0.4–2 mg/kg) intake are reported also in several studies [44,45,46,48]. ASD Omani children had statistically (*p* < 0.05) higher homocysteine levels (20.1±3.3 μmol/L) in respect to controls (9.64 ± 2.1 μmol/L) [47]. Additionally, the homocysteine levels in ASD children were considerably higher compared to normal reference values (5–15 μmol/L), whereas serum concentrations of folate and Vitamin B12 were much below the values defined as deficiency levels (3.0 μg/L and < 250 pg/mL, respectively) for these nutrients [47]. 

Regarding the studies that have investigated the folate levels in ASDs children, Shoffner in his cohort study examined CSF 5-MTHF concentrations in 67 ASD diagnosed children and did not observe any significant correlation between CSF 5-MTHF and autism symptoms. CSF 5-MTHF levels less than 40 nmol/L were observed in 11 of 67 children but only in one of two repeated CSF evaluations. Findings showed that CSF 5-MTHF levels differ significantly over time in an unpredictable way, indicating a biological variability, and have no relationship with typical clinical features of autism [50].

In a recent multi-center study performed in China and involving 1300 ASDs diagnosed children, Li et al., correlated the serum folate levels with the clinical aspects and the neurodevelopmental levels of different age groups; the authors found serum folate levels lower than in normally developing children. Furthermore, the folate serum concentration in the ASD children is interestingly associated with the neurodevelopmental level [51].

### 3.5. Studies on MTHFR Polymorphisms and ASDs

On the other hand, an important consideration clarified the role of the MTHFR C677T polymorphism and ASDs (Table 3) [53,55,56,59]. A population-based case-control study in Chinese Han assessed the frequency of genotype MTHFR 677TT in 372 children. The frequency of these genotype in children with autism (16.1%) was significantly higher (odds ratio [OR] = 2.04; 95% confidence interval [CI] = 1.07, 3.89; *p* = 0.03) than those in controls (8.6%), suggesting that MTHFR C677T is a risk factor for autism in children of this population [52].

El-Baz investigated C677T and A1298C polymorphic genotypes of the MTHFR gene showing a significant association between severity and occurrence of autism [60]. Patients had highest heterozygosity for A1298C polymorphism (41.9%), with respect to mutant genotype CC (35.5%) and normal AA (wild) type (22.6%). Allele C appeared in patients more than in controls (56.45% vs. 11.54%) (*p* < 0.001). Patients had highest heterozygosity for C667T polymorphism (48.4%) compared to wild type genotypes CC (38.7%) and mutant genotypes TT (12.9%). Allele T was detected more in patients than controls (31.10% vs. 5.13%) (*p* < 0.00). Furthermore, heterozygosity for CT and AC genotypes were detected equally (46.2%) among patients with severe autism (according to childhood autism rating scale).

A Brazilian case-control study showed no significant changes between cases and controls (*p* = 0.72) in terms of frequency of T allele (0.38 for the case group and 0.35 for the control group) (*p* = 0.77) [54]. Concordant observations were made in addition by Sener and Zhang [57,58]. Moreover, the authors suggested a replication of the studies with a larger well-characterized scale. The study of 529 case–parent trios vs. 566 neurotypical controls showed that maternal genetics/ epigenetics may affect fetal predisposition to autism [61]. Plasma homocysteine, adenosine, and AdoHcy resulted as significantly elevated between autism mothers, corresponding to reduced methylation capacity and significant DNA hypomethylation (*p* < 0.001). Further analysis revealed a significant increase in the reduced folate carrier (RFC1) G allele frequency among case mothers, but not among fathers or affected children, determining a significant increase of the risk of autism.

### 3.6. Studies on the Frequency of Serum FRAA in ASD Children

Table 4 contains the results obtained in three human studies investigating the frequency of FRAA in ASD children [31,62,63]. The first cohort study includes 40 ASDs children and 42 gender-age matched typical development (TD) children, age range of 2 to 6 years. The author found that serum FRAA concentration in ASD children was higher than in TD children (138.61 ±373.27 ng/mL vs. 37.68 ±71.54 ng/mL, *p* = 0.09829), where 77.5% (31/40) of children with ASDs and 54.8% (23/42) of TD children were positive for serum FRAA (*p* = 0.03746) [62]. In the second study, Ramaekers [31] et al., assessed additional parameters analyzing serum and CSF folate, serum vitamin B12, homocysteine and amino acids concentration, serum, and CSF FRAA and FR1 and FR2 genes. Results highlighted that all 25 age-matched controls were negative for FRAA, while 19 out of the 23 patients with low CSF 5MTHF had serum FRAA with a mean value of 1.05 pmol FR blocked/mL serum (range: 0.1–4.19). Furthermore, treatment with folinic acid (1–3 mg/kg/day) in these patients led to partial or complete recovery from ASDs in early diagnosed cases (before the age of three years old). An improvement in verbal communication was found by Frye et al. [63] in his cohort of non-syndromic ASD children through folinic acid treatment.

### 3.7. Summary of the Results Reported by Animal Clinical Studies Included in the Systematic Review

In Table 5, the principal findings of the nine animal clinical studies are reported. Animal studies showed how maternal periconceptional deficit of folate in rats provokes behavior alterations in the offspring relevant to the autistic-like phenotype [64,65,66]. Analysis of gene expression in the cerebellum of offspring mice from 8 to 10-week-old pups revealed that the expression pattern of a significant number of genes were found to be altered by ≥2.5 fold at a significance of *p* > 0.05 after exposure to 20 mg/kg high maternal FA (HMFA) diet during gestation, suggesting a dysregulated expression of several genes in the cerebellum of both male and female pups [67].

Likewise, studies on mice with a Balb/cAnNCrlBR background and heterozygous MTHFR-knockout indicated that maternal and offspring MTHFR deficiency increased the risk for an ASD-like phenotype in the offspring [27,68]. Orenbunch observed a reduced risk of ASD-like behavior in MTHFR-deficient mice supplemented with one-carbon nutrients prenatally (FA 9 mg/mL, betaine 2%, choline 2%) [27]. Specifically, among offspring of MTHFR+/− dams, prenatal diet supplementation was protective against ASD-like symptomatic behavior compared to the control diet with an odds ratio of 0.18 (CI:0.035, 0.970). Additionally, a change in the cerebralcortex of the proportions between betaine/choline and SAM/SAH were correlated with behavior similar to ASDs. Males had an altered ratio of the glutamate receptor subunits GluR1/GluR2 with respect to NR2A/NR2B. Moreover, symptomatic mice with ASD-like behavior had lower levels of GABA pathway proteins (GAD65/67 and VGAT) [27].

Regarding the frequency of FRAA on ASDs only two studies, conducted in 2016 in rat models, suggested severe behavioral and cognitive changes mirroring ASD symptoms when exposed to FRα antibodies during gestation [69,71].

In a recent paper, Chu et al., supplemented the diet of male and female mice prior to mating, during pregnancy and lactation with moderate and high FA doses (2.5 and 10 times the normal dietary intake respectively) and evaluated the effect to offspring at weaning. Interestingly, the authors found that behavioral abnormalities were more evident in mice fed with moderate FA doses than in those treated with higher FA doses. However, both moderate and high FA supplementation modified the cerebral gene expression in offspring at weaning, but these FA doses were not sufficient to induce autism-like behavior [70].

## 4. Discussion

### 4.1. Summary of Evidence

The results emerging from studies evaluating the relationship between FA and ASD risk are not always consistent: in some of them, the maternal FA supplementation results in a reduced ASD risk [34,35,36,37,38,39,40,41,42,43,73,81,82,83]; other studies do not confirm these positive results, finding an enhanced risk following the supplementation [72,84,85,86,87]; furthermore, some authors did not obtain any association between folate intake and ASD risk [34,74,88], or not satisfactory conclusions about the utility of folate supplementation [76].

The relationship between folate and ASDs is extremely complex overall because the low folate-dependent DNA hypomethylation can be due not only to a reduced intake but also to an altered folate metabolism [14], an example of which is the debated epigenetic role of MTHFR polymorphisms (C677T and A1298C) in the DNA methylation. MTHFR C667T polymorphism shows regional and ethnic variations; additionally, the frequency of the T allele was higher in ASD offspring consequently identified as a risk factor. Cytosolic serine hydroxyl methyl transferase (SHMT1 C1420T), MTHFR A1298C, methionine synthase reductase (MTRR A66G), and methionine synthase (MS A2756G) were assessed as well. This issue has been investigated in a meta-analysis by Pu et al. [75], who interestingly found that MTHFR C677T can be associated with an increased risk of ASDs, but only in countries without mandatory FA fortification; these results emphasize the potential relevance of the FA prenatal supplementation in modulating the ASDs risk in the presence of MTHFR C677T polymorphism [81].

Another principal observation of this review was the frequency of FRAA in serum or CSF and their association with ASDs. Serum FR autoimmunity appears to represent an important factor in the pathogenesis of reduced folate transport to the nervous system among children with early-onset low-functioning autism, associated with or without neurological deficits. The elevated presence of FRAA in ASD offspring could reduce folic acid uptake in the choroid plexus, explaining the reduced CSF folate levels, representing a useful screening biomarker for ASDs [31]. Early detection of FRAA may be a key factor in the prevention and therapeutic intervention among this subgroup of patients with autism. We further observed that clinical evaluation and confirmation in large sample sizes of the results obtained is necessary, considering additionally to investigate whether FA intake can improve the syndrome of FRAA positive children with ASDs. In this subgroup of ASDs children, in some studies [63,89], the treatment with folinic acid has been successfully used; folinic acid, as a reduced form of FA, can enter the nervous central system through the blood–brain barrier by using the reduced folate carrier when the FR is unavailable because of the presence of FRAA.

According to an interesting recent study [80], also the maternal post-delivery high concentrations of folate could be the consequence of an altered mechanism of transport of the micronutrient across the placenta, depending on a higher prevalence of FRAAs or of a mutation in RFC, as shown in mothers of ASD offspring. Then, according to this author, FRAAs could induce a condition of folate deficiency in the fetus, even with a normal maternal folate status.

Only a few studies measured vitamin B12, homocysteine levels, and glutathione levels, while none of them analyzed red blood cell folate. The relevance of vitamin B12 in the onset of autism and ASDs can be strictly connected to its role in DNA methylation, the epigenetic regulatory process known to be relevant to brain development [90]. Amanat indicated that serum homocysteine levels were significantly higher in autistic children compared to controls, and significantly lower serum folate and vitamin B12 levels were observed in autistic children compared to controls [47]. Intervention treatment with FA resulted in improvement of ASD-associated behaviors and metabolic profile in autistic children, especially in those with early established diagnosis [44]. These findings suggest a recovery of the methyl groups transfer via the methionine cycle lowering homocysteine levels, redirecting it to the transsulfuration pathway as well to avoid brain dysfunction via oxidative damage and abnormal DNA methylation. Clearly, methyl donors are functionally dependent of one another; therefore, further studies should incorporate homocysteine and vitamin B12 concentration measurements into their exposure evaluations during the periconceptional period and early pregnancy to better explicate the association of folate with ASDs.

In studies that have evaluated the risk of ASD, different doses of FA maternal supplementation have been considered, in addition to different windows of exposure to the vitamin supplementation.

This lack of homogeneity gave rise to an interesting debate about the potential enhanced risk of ASDs following high dosage of FA intake pre or during pregnancy; most of the studies, performed both in humans and in animals, emphasizing the warning in the use of high FA dosages, focused on the presence of circulating, and very likely detrimental, unmetabolized FA [14,15,72,79,91], usually associated with clinical conditions such as the reduced cytotoxicity of natural killer cells in animal studies [92], neurological and cognitive disorders [93], or cancer.

Several neurological disorders in brain development have been observed in some animal studies following maternal supplementation with large doses of FA: synaptic defects [94], dysregulation of gene expression [67], and effects on brain DNA methylation [84]. Synthetic FA is reduced to tetrahydrofolate by dihydrofolate reductase (DHFR), whose activity in humans is slow and can be inhibited by higher concentrations of the same FA [95]; however, unmetabolized FA, usually is not present in serum in the case of FA supplementation not exceeding 400 ug/day [96].

A lower concentration of 5-MTHF in the cell, a reduced transformation to methionine, and an altered methylation process can be the consequences of high concentrations of unmetabolized FA; in this regard, several animal studies have interestingly shown both gene-specific hypermethylation and DNA hypomethylation [77,97,98] in animals fed with higher dosages of FA, and a different grade of modulation of the global DNA methylation has been recently proposed also by Chu et al. [70]. Further investigations are, however, requested in humans.

A detrimental effect of unmetabolized FA on neural districts during neurological development might be then hypothesized [86,99,100].

Unmetabolized FA can arise not only from FA intake, pre or during pregnancy, at higher dosages than 1 mg/d but, according to a recent study [85], it could depend also on an impaired function of the one-carbon metabolism with or without vitamin B12 involvement [78]; another consideration can be made in this regard: the exact critical time windows during which the neurological development can be influenced by the one-carbon metabolism are not yet fully defined.

As previously underlined, folate is currently and successfully used in early pregnancy to reduce the risk of NTDs at a dosage, usually of 400 ug/day or up, at least to the end of the first trimester. However, many women at increased risk for NTDs, or at risk for recurrence of NTDs, are advised to intake FA dosage higher than 1 mg/day.

Further studies then aimed to investigate the real responsibility, if any, of unmetabolized FA in the onset of ASDs are absolutely necessary, especially because the presence of high levels of FA in the maternal blood is not an extraordinary finding, particularly in countries where food fortification with FA is mandatory.

### 4.2. Limitations

Some of the limitations of the studies are the heterogeneity of FA supplementation, the duration of use of these supplements, and the residence of the study participants in regard to the approved nutritional fortification. Moreover, the small sample size relative in some studies can be an additional limitation.In order to evaluate the genetic influence on ASDs in offspring only a few studies reported data from the potential presence of paternal polymorphisms in one-carbon metabolism.

## 5. Conclusions

In summary, this systematic review aimed to clarify the association between FA and ASDs, taking into consideration biological, genetic, and epidemiological evidence as important complex mechanisms required for maintaining optimal folate levels. One of the main concerns emerging from the studies is that nutritional fortification with folic acid present in some countries can cause higher maternal blood folic acid levels, potentially leading to detrimental circulating of unmetabolized FA, especially if associated with FA supplements pre or during pregnancy. Another concern not fully defined is the exact critical time window during which the neurological development can be influenced by the one-carbon metabolism. Further research taking in consideration the limitations of the current studies reported in this systematic review should be carried out to give a clear overview of the correlation between FA and ASDs.

## Figures and Tables

**Table 1:** Studies on the association between maternal FA supplementation and the risk of ASDs in offspring.

No	Study	Type of Study	Sample Size	Supplement Used	Period of Intake	ASDs Cases	Study Outcome Parameters	Outcomes
1	Virk J et al., (2016) [34]	Population-based cohort study	n = 35,059 women	FA and/or multivitamin supplements (containing at last 400 μg of FA)	4 weeks prior to 8 weeks after pregnancy	552 (1.6%)	Autism and Asperger’s syndrome. Pervasive developmental disorder—not otherwise specified (PDD-NOS)	No association has been found between early folate or multivitamin intake for autism spectrum disorder [34].
2	Levine SZ et al., (2018) [35]	Case-control cohort study	n = 45,300 children	FA and/or multivitamin supplements	Before but not during pregnancy/during but not before pregnancy/before and during pregnancy from 4 weeks before and 8 weeks into pregnancy2 years before pregnancy	572 (1.3%)	Risk of ASDs in offspring	Maternal exposure to FA and multivitamin supplements before and during pregnancy is associated with a reduced risk of ASDs in the offspring compared with the offspring of mothers without such exposure [35].
3	Surén Pet al., (2013) [36]	Cohort study	n = 85,176 children	Multivitamin supplements (400 μg of FA) fish oil and FA	4 weeks before to 8 weeks after the start of pregnancy	270 (0.32%)	Autistic disorder risk. Asperger syndrome. PDD-NOS	Maternal use of supplemental folic acid before conception and early in pregnancy was associated with a lower risk of autism spectrum disorders in children [36].
4	DeVilbiss EA et al., (2017) [37]	Population- based cohort study	n = 273,107 mother–child pairs	Multivitamins supplements FA supplements (400 μg)	First trimester (400μg/day)	158 (0.26%) in the maternal multivitamin use group 430 (0.48%) in the no-nutritional supplementation use group	Risk of ASDs with and without intellectual disability in offspring	Maternal multivitamin supplementation during pregnancy may be inversely associated with ASDs with intellectual disability in offspring [37].
5	Raghavan R et al., (2018) [38]	Cohort study	n = 1257 mother–infant pairs	Multivitamin Supplements	During pregnancy≤2 times/week3–5 times/week>5 times/week	86 (6.8%)	Risk of ASDs in offspring.Maternal B_12_ levels 2–3 days after birthMTHFR genotype	The results show that moderate intake (3–5 times/week) of multivitamin supplements during pregnancy is associated with decreased risk of ASDs in offspring. Low (≤2 times/week) and high (>5 times/week) supplementation was associated with increased risk of ASDs. There was a “U” shaped relationship between maternal multivitamin supplementation frequency and ASDs risk [38].
6	Schmidt RJ et al., (2019) [39]	Cohort study	n = 332 children and theirn = 305 mothers	FA (600 μg) and iron (27 mg) supplements	First month of pregnancy 6 months before and each month during the pregnancy	18 (14.1%) following mother supplementation and 37 (32.7%) from mothers receiving no supplementation	ASD recurrence in siblings of children with ASDs in high-risk families	Maternal prenatal vitamin intake during the first month of pregnancy may reduce ASDs recurrence in siblings of children with ASDs in high-risk families [39].
7	Steenweg-de Graaff J et al., (2015) [40]	Population-based birth cohort study	n = 5591 mothers of single live-born neonates	FA supplements	Preconceptional within the first 10 weeks of pregnancy After the first 10 weeks of pregnancy	3893 (70%)	Maternal plasma folate concentrations at 13 weeks of gestation. Autistic traits in the offspring at the age of six years	Maternal folate was not associated with autistic traits in the offspring. In contrast, prenatal folic acid use was associated with less child autistic traits [40].
8	Nilsen RM et al., (2013) [41]	Population-based cohort study	n = 507,856 Medical Birth Registry of Norway nationwide population children n = 89 836 MoBa cohort sample children	FA supplements	Prenatal exposure	234 (0.26%) in the cohort and 2072 (0.41%) in the nationwide population	Specialist-confirmed diagnosis ASDs.Estimated the risk of ASDs associated with: primipara pregnancy (no, yes), prenatal folic acid use (no, yes), prenatal smoking (no, yes), low birth weight (no, yes), preterm birth (no, yes), offspring sex (female, male), and caesarean section history (no, yes).	Was observed that maternal prenatal folic acid supplement use was associated with a 14–17% adjusted risk reduction for ASDs [41].
9	Christian P et al., (2010) [42]	Cohort study	n = 676 children	FA (400 μg) FA and iron (60 mg) FA, iron and zinc (30 mg) FA, iron, zinc and vitamins D (10 μg), E (10 mg), B1 (1.6 mg), B2 (1.8 mg), B6 (2.2 mg), B12 (2.6 μg), C (100 mg), and K (65 μg); niacin (20 mg); copper (2.0 mg); magnesium (100 mg), with 1000-μg vitamin A	early pregnancy for 3 months postpartum	N.S	Estimation of children’s intellectual functioning:universal nonverbal intelligence test (UNIT); tests of executive function, including go/no-go, the Stroop test, backward digit span test; movement assessment battery for children (MABC); finger-tapping test	Maternal prenatal FA and iron supplementation was associated with better test scores in offspring at 7 to 9 years compared to the control group with only vitamin A supplementation [42].
10	Roth C et al., (2011) [43]	Cohort study	n = 38,954 children	FA and/or multivitamin supplements	4 weeks before to 8 weeks after conception	204 (0.5%) severe language delay and 1290 (3.3%) moderate language delay	Risk of child with severe language delay. Assessment of severe language delay and motor delay	Maternal FA use was associated with a reduced risk of severe language delay in offspring at age 3 years. Instead, no significant association with motor skills delay risk was found [43].

**Table 2:** Studies of folate supplementation or folate levels in ASDs diagnosed children.

No	Study	Type of Study	Sample Size	Age	Supplement Used	Period of Intake	Study Outcome Parameters	Outcomes
1	Sun Cet al., (2016) [44]	Open-Label Trial	n = 66 ASDs children	4.5 ± 1.1 years old	FA supplements (400 μg)	Two times/daily3 months	Plasma levels of: FA homocysteine glutathione metabolism before and after treatment. Improvement of autism symptoms	Intervention improved autism symptoms towards sociability, cognitive verbal/preverbal, receptive language, and affective expression and communication. Furthermore, this treatment also improved the concentrations of folic acid, homocysteine, and normalized glutathione redox metabolism [44].
2	Moretti P et al., (2005) [33]	Single study subject	n = one girl	6 years old	Folinic acid supplements (0.5 mg/kg)	One time/daily doubled after 2 weeks1 year	CSF levels of 5-methyltetrahydrofolate (5-MTHF), pterins and neurotransmitter metabolites	Treatment with folinic acid corrected CSF abnormalities and improved motor skills. The evaluation showed that despite improvement of motor skills and parental reports of increased responsivity, her cognitive, language, and socialization skills remained delayed [33].
3	James SJ et al., (2009) [45]	Open-label trial	n = 40 ASDs children	2–7 years old	Methylcobalamin (75 μg/kg) Folinic acid (400μg)	2 times/week (methylcobalamin) 2 times/day (folinic acid) 3 months	Plasma levels of: methionineSAMSAHSAM:SAH ratio. Homocysteine, cysteine, cysteinylglycine, tGSH, fGSH, GSSG	The significant improvements observed in transmethylation metabolites and glutathione redox status after treatment suggest that targeted nutritional intervention with methylcobalamin and folinic acid may be of clinical benefit in some children who have autism [45].
4	Kałużna-Czaplińska J et al., (2011) [46]	Case-control study	n = 30 ASDs children vs.21 non ASDs children	4–11 years old	FA supplements (400 μg) Vitamin B_6_ (200 mg) Vitamin B_12_ (1.2 μg) (sugar-free diet)	3 months	Urine homocysteine levels in ASDs children before vitamin supplementation and 3 months after	The study showed a significant improvement in sleep and gastrointestinal problems compared with the placebo group. The intake of vitamins B_6_ and B_12_, together with folic acid, was found to be more effective in lowering the levels of urinary homocysteine than the intake of vitamins B_6_ and B_12_ alone [46].
5	Amanat A et al., (2011) [47]	Case-control study	n = 40 ASDs children vs 40 TD children of their age and gender matched controls	3–5 years old	No supplements	N.A	Fasting serum homocysteine levels. Serum folate and vitamin B_12_ levels. Follow up time: December 2009–August 2010	The results indicated that mean serum Hcy levels were significantly higher in autistic children as compared to controls. Significantly lower serum folate and vitamin B_12_ levels were observed in autistic children as compared to controls. The levels of homocysteine in autistic children were also much higher as compared to normal reference values (5–15 μmol/L) [47].
6	Guo M et al., (2020) [48]	Clinical Trial	n = 274 ASDs children vs. 97 TD age-matched children	4.24 ± 1.20 years old	No supplements	N.A	Vitamin and mineral concentrations in ASDs and TD children. Influence of vitamin and mineral status on the Autism Behavior Checklist (ABC) score, Social Responsiveness Scale (SRS), and correlation analysis between the Gesell Developmental Scale (GDS) scores	Results showed a significant insufficiency of vitamin and mineral, especially of folate and vitamin D, in ASDs children. The analysis correlated these lower nutrient levels with ASDs traits [48].
7	Adams JB et al., (2018) [49]	Randomized controlled single-blind study	n = 67 children and adults with ASDs from Arizona vs.50 non-sibling neurotypical controls of similar age and gender	3–58 years old	Special vitamin/mineral supplements (~600 mcg FA)Essential fatty acids (omega-3 and omega-6 fatty acids).Carnitine supplement	12 months	Blood and urine levels of: vitamins, biomarkers of vitamin status, minerals, plasma amino acids, plasma glutathione, neurotransmitters, and biomarkers of oxidative stress, methylation, sulfation and energy production	There was a significant improvement in nonverbal intellectual ability in the treatment group compared to the non-treatment group based on a blinded clinical assessment. Based on semi-blinded assessment, the treatment group, compared to the non-treatment group, had significantly greater improvement in autism symptoms and developmental age. The treatment group had significantly greater increases in EPA; DHA; carnitine; and vitamins A, B_2_, B_5_, B_6_, B_12_, folic acid, and coenzyme Q_10_ [49].
8	Shoffner J et al., (2016) [50]	Cohort study	n = 67 children with a diagnosis of DSM-IV-TR autistic disorder at last one lumbar puncture (LP)	2–6 years old	No supplements	N.A	5-methyltetrahydrofolate concentration in CSF and blood samples (follow up time: 30 ±8 months).	CSF 5-MTHF levels vary significantly over time in an unpredictable fashion and do not show a significant relationship to typical clinical features of autism [50].
9	Li Q et al., (2021) [51]	Multi-center study	n= 1300 ASDs children vs. 1246 TD children	<7 years old	No supplements	N.A	Serum folate levels. Effect of serum folate level on symptoms assessed with ABC, SRS, and Childhood Autism Rating Scale (CARS)	The results showed that serum folate levels were lower in ASDs children comparing to the levels found in typically developing children. Moreover, the author underlined the necessity to evaluate folate status in children with ASDs aged three and under [51].

**Table 3:** Studies on the role of MTHFR Gene C677T Polymorphism in ASDs risk.

No	Study	Type of Study	Sample Size	Age	Aim of Study	Study Outcome Parameters	Outcomes
1	Guo T et al., (2012) [52]	Population-based case-control study	n = 186 ASDs children vs. 186 control children	8.1 (±4.3) years old	Role of the MTHFR C677T polymorphism on the autism risk in the population	Frequency of genotype MTHFR 677TT in children	The frequency of genotype MTHFR 677TT in children with autism was significantly higher than those in controls. This study suggested that MTHFR C677T is a risk factor of autism in Chinese Han children [52].
2	Goin-Kochel RP et al., (2009) [53]	Exploratory genotype-phenotype correlations study	n = 147 ASDs children	7.9 years old	Potential differences among MTHFR genotypes for specific behaviors	Blood samples genotyped for the MTHFR 677C-T polymorphism	The results provide preliminary evidence supporting a relationship between MTHFR 677C-T genotype and specific behaviors among children with autism [53].
3	Santos PAC dos et al., (2010) [54]	Case-control study	n = 151 ASDs children vs. 100 healthy control children	<3 years old	Association between C677Tpolymorphism and ASDs	ADI-R criteria used for the evaluation of patient’s behavior genotype distribution of the MTHFR C667T polymorphism	The frequency of the T allele was 0.38 for the case group and 0.35 for the control group (*p* = 0.77). The genotypic distribution did not show significant differences between cases and controls (*p* = 0.72) nor association between the T allele and selected behaviors [54].
4	Mohammad NS et al., (2016) [55]	Case-control study	n = 138 ASDs children vs. 138 non-autistic children of matched age	(4.4±1.7) years old vs. (4.4±1.6) years old	Development of an artificial neural network (ANN) model from the data of 138 autistic and 138 non-autistic children using GCPII C1561T, SHMT1 C1420T, MTHFR C677T, MTR A2756G, and MTRR A66G as the predictors of autism risk	Genetic analyses:GCPII C1561T, SHMT1 C1420T, MTHFR C677T, MTR A2756G, and MTRR A66G as predictors of autism risk.Plasma homocysteine determination	Genetic polymorphisms of the folate pathway were moderate predictors of autism risk. MTHFR C677T and hyperhomocysteinemia have been identified as risk factors for autism worldwide. Synergistic interactions between MTHFR C677T and MTRR A66G increase homocysteine [55].
5	Ismail S et al., (2019) [56]	Case-control study	n = 78 ASDs children vs. 80matched healthy control children	3–6 years old	Investigate the association of MTHFR gene rs1801133 (C677T) variant among ASDs children	Full clinical and radiological examinations DNA genotyped for MTHFR genetic variant (C677T)	MTHFR (C677T) allele frequency was found to be higher significantly in ASDs cases compared with non-autistic children. Additionally, there was a higher distribution of combined CT + TT genotypes among autistic patients with consanguinity and family history of psychological disease [56].
6	Zhang Z et al., (2018) [57]	Case-control study	n = 201 ASDs children vs. 200 healthy control children	-	Association between childhood ASDs and single-nucleotide polymorphisms (SNPs) in genes involved with vitamin B12 and folate metabolism	Genotypes of transcobalamin 2 (TCN2) rs1801198, methionine synthase (MTR) rs1805087, methionine synthase reductase (MTRR) rs1801394, and methylene tetrahydrofolate reductase (MTHFR) rs1801133 were examined	Results showed no association of all examined single-nucleotide polymorphisms SNPs with childhood ASDs and its severity [57].
7	Sener EF et al., (2014) [58]	Cohort study	n = 98 ASDs children vs. 70 age and sex-matched non-autistic children	≦3 years old	Investigate the possible effect of C677T polymorphisms in a population cohort	DNA tested for MTHFR C677T polymorphism	MTHFR 677T-allele frequency was found to be higher in autistic children compared with non-autistic children, but it was not found statistically significant [58].
8	Mohammad NS et al., (2009) [59]	Population study	n = 138 ASDs children vs. 138 age and sex matched nonautistic children	2–10 years old	Investigate whether genetic polymorphisms are the underlying causes for aberrations in folate pathway reported in autistic children	DNA tested for five genetic polymorphisms: cytosolic serine hydroxyl methyl transferase (SHMT1 C1420T), methylene tetrahydrofolate reductase (MTHFR C677T, and MTHFR A1298C), methionine synthase reductase (MTRR A66G), methionine synthase (MS A2756G)	MTHFR C677T is a risk factor, whereas MTRR A66G and SHMT C1420T polymorphismsreduce the risk for autism. MTHFR A1298C acts additively in increasing the risk for autism [59].
9	El-Baz F et al., (2017) [60]	Case-control study	n = 31 ASDs children vs. 39 children normal control group	4.5 ± 2 years old	Identification of C677T and 1298AC polymorphic genotypes of MTHFR gene among a sample of children with autism	Identification of C677T and 1298AC polymorphic genotypes of MTHFR gene	There is a significant association between severity and occurrence of autism with MTHFR gene polymorphisms C677T and A1298C. Further studies are needed on a larger scale to explore other gene polymorphisms that may be associated with autism to correlate the genetic basis of autism [60].
10	James SJ et al., (2010) [61]	Population-basedcase-control study	n = 529 case-parent triosvs. 566 TD controls	3–10 years old	Investigate the frequency of common functional polymorphisms in the folate pathway	Allele frequencies of MTHFR C677T, MTHFR A1298C, TCII C776G, or MTRR A66G among mothers, fathers, or affected child compared to population controls. Determination of percent 5-methylcystosine/ total cytosine in DNA plasma transmethylation metabolites genetic relative risk and likelihood ratio test, transmission disequilibrium test, maternal plasma transmethylation metabolites and plasma folate concentrationslobal DNA methylation density and RFC1genotype association among Arkansas mothers	The results showed a significant increase in the reduced folate carrier (RFC1) G allele frequency among case mothers but not among fathers or affected children. Subsequent log linear analysis of the RFC1 A80G genotype within family trios revealed that the maternal G allele was associated with a significant increase in risk of autism, whereas the inherited genotype of the child was not.Results suggest that the maternal genetics/epigenetics may influence fetal predisposition to autism [61].

**Table 4:** Studies on the frequency of serum FRAA in ASDs children.

No	Study	Type of Study	Sample Size	Treatment	Age	Study Outcome Parameters	Outcomes
1	Ramaekers V et al., (2007) [31]	Case-control study	n = 25 patients early-onset low-functioning autism vs. 25 controls age match	Folinic acid supplementation dose of 1–3 mg/kg/day3–6 months following	2.8–12.3 years old	Serum and cerebrospinal fluid (CSF) folate level analysis and autoantibodies against FRs assay. Serum FA, vitamin B_12_, homocysteine, and amino acids concentrationFR1 and FR2 genotyping	Reduced CSF folate levels were observed in 19 of these 23 patients. Oral folinic acid supplementation led to normal CSF 5-MTHF levels and partial or complete clinical recovery after 12 months. Serum FR autoimmunity appears to represent an important factor in the pathogenesis of reduced folate transport to the nervous system among children with early-onset low-functioning autism associated with or without neurological deficits [31].
2	Zhou J et al., (2018) [62]	Cohort study	n = 40 ASDs children vs. 42 gender and age matched TD children	No supplementation	Younger than 14 years old (2–6 years old)	Serum FRAA concentrations	Serum FRAA are more prevalent in children with ASDs than in TD children, suggesting that children with ASDs may have defects in folic acid absorption that play a role in the onset of ASDs [62].
3	Frye RE et al., (2018) [63]	Double-blind randomized placebo-controlled parallel study	n = 48 ASDs children	Folinic acid (2 mg/kg) (maximum 50 mg) per day for 12 weeks	±7 years old	Improvement of verbal communication. Development of language and communication skills	Improvement in verbal communication was significantly greater for the participants on folinic acid compared with participants on placebo with a medium-to-large effect size, particularly in those participants who were positive for FRAAs [63].

**Table 5:** Animal clinical studies on the role of folic acid in ASDrisk.

No	Study	Type of Animal	Treatment Used	Study Outcome Parameters	Sample Size	Outcomes
1	Degroote S et al., (2018) [64]	Female Wistar rat	0.2 mg/kg FA	Blood homocysteine levels. Behavioral alterations relevant to autism-like in offspring	n = 12 female, n = 8 male	Maternal periconceptional deficit in folate provokes alterations in the behavior of offspring relevant to the autistic-like phenotype [64].
2	Barua S et al., (2016) [65]	Female C57BL/6J Mice	2 mg/kg control diet FA (CD) 20 mg/kg high maternal FA (HMFA)	Expression of genes in the cerebral hemispheres (CHs) of 1-day-old pups, FA concentration in CH Tissue	n = 12 CD female, n = 12 HMFA female	The results showed that HMFA supplementation alters offsprings’ CH gene expression in a sex-specific manner. These changes may influence infants’ brain development. In addition, it was also found that HMFA had no impact on global DNA methylation levels of the offspring epigenome [65].
3	Barua S et al., (2015) [67]	C57BL/6 J mice	2 mg/kg FA,20 mg/kg FA	Expression of genes in the cerebellum of offspring from day old pups	n = 6,n = 6	Results revealed that exposure to the higher dose FA diet during gestation dysregulated expression of several genes in the cerebellum of both male and female pups [67].
4	Kezurer N et al., (2013) [66]	Mice on a Balb/cAnNCrlBR backgroundMTHFR heterozygote	Mild neonatal stress (25 μL PBS at postnatal days 4–10)	Offspring genotyped of DNA isolated from toe clips. Reflex analysis and newborn development. Reflex development in newborn mice. Anxiety-related behavior.Adult behavior. Corticosterone levels	n = 97 mice (7–14 in each group) were tested	Overall, the results support an interaction between mild neonatal stress, the MTHFR genotype, and sex [66].
5	Orenbuch A et al., (2019) [27]	Mice with a Balb/cAnNCrlBR backgroundheterozygous for the Mthfr-knockout	9 mg/mL FA in the enriched food group	Tissue concentrations of C1 metabolites in the liver, cerebral cortex, and basal forebrainMthfr/ Genotypingbehaviors	5 groups of mice from each sex	The study suggests that MTHFR deficiency can increase the risk of ASD-like behavior in mice and that prenatal dietary intervention focused on MTHFR genotypes can reduce the risk of ASDs-like behavior. Findings emphasize the critical role of in utero C1 metabolism in developmental trajectories that lead to the presentation of autistic behavior. Aberrations in both the GABAergic and glutamatergic pathways suggest that Mthfr deficiency is linked to deleterious alterations in the basal cortical circuit activities in the affected mice [27].
6	Sadigurschi N et al., (2019) [68]	Mice on a Balb/cAnNCrlBR backgroundheterozygote Mthfr-KO mice	No treatment	Genotyping of DNA isolated from toe clips. Immuno-fluorescence analysis of brain tissue. Morphogenic and behavioral assessments	N.S (not specified)	The study provides evidence for the profound impact of a genetic deficiency in the MTHFR gene on the induction of autistic features. In the mouse model of ASDs, this deficiency directly regulates metabolite availability and indirectly controls the environment of the developing embryonic brain [68].
7	Sequeira JM et al., (2016) [69]	Long Evans hooded Rat	Normal diet containing 2 mg/kg FAFRα- Ab at a dose of 4μg/embryo mixed intra-peritoneal (IP) injection FRα-Ab (35μg per pup per day) on post-natal days (PND) 10, 11 and 12 (GST-PRW group). Single dose of antibody at 4.0 μg/embryo on GD8. Antibody (50μg in 0.2ml rat serum IP) on PND 26, 27, and 28 (POW group)	Behavioral tests investigated the effect of exposure to FRα antibodies (Ab) during gestation (GST), the pre-weaning (PRW), and the post weaning (POW) periods on learning and behavior in adulthood	N.S	Deficits in rats exposed to Ab during gestation and pre-weaning (GST+PRW) included indications of increased levels of anxiety. None of these rats learned the active place avoidance task, indicating severe learning deficits and cognitive impairment. Similar but less severe deficits were observed in rats exposed to Ab during GST alone or only during the PRW period, suggesting the extreme sensitivity of the fetal as well as the neonatal rat brain to the deleterious effects of exposure to Ab during this period [69].
8	Chu D et al., (2019) [70]	ICR mice	NIH-31 open formula diet containing 2 mg /kg FA.Deionized water for 1week containing: no FA (control) 3.75 mg/L FA (MFA) 22.5 mg/L FA (HFA)	Behavioral testsRNA sequencing for postnatal day 21 mice brain. Expression of genes in adult mice brain 5 months postpartum of each group.Body weight of the offspring for 5 months	n = 6 male, n = 12 female	Results demonstrated a change in gene expression profile in weaning mice during early life FA supplementation. Long-term behavioral effects were more evident in adult male mouse offspring in a dose-sensitive mode [70].
9	Desai A et al., (2017) [71]	Rat	Normal diet containing 2 mg/kg FAFRα Ab at a dose of 4 or 12 μg per embryo in 1 mL normal rat serum was administered by (IP) injection1 mg of folinic acid (GD7–GD12) IP and/or 0.5 mg	Folate uptake and FRα Ab localization studies. Behavioral tests	50	Findings suggest severe behavioral and cognitive changes mirroring ASD symptoms following gestational Ab exposure in a rat model and protection afforded by folinic acid and dexamethasone treatment [71].

**Table 6:** An overview of all reviews, systematic reviews, and metanalysis included in the study.

No	Study	Type of Study	Aim of Study	Parameters Assessed in the Study	Outcomes
1	DeVilbiss EA et al., (2015) [14]	Review	Overview and summaries of the folate role in neurodevelopmental disorders; relationship between maternal folate and ASDs	Maternal folate and autism spectrum disorders and related traits. Self-reported maternal folate and autism spectrum disorder traits. Maternal folate biomarker and autism spectrum disorder traits. Folate supplementation and ASDs	Inconclusive evidences underline the need for future studies of maternal folate status during the pre- and peri-conceptional periods. In addition, an incorporation of genetic data could complete better these assessments [14].
2	Wiens D et al., (2017) [72]	Review	Examination of folic acid (FA) effects on neuronal development from tissue culture experiments, understanding ASDs metabolic causes and alternative folinic acid treatment	Unmetabolized FA neural development metabolic abnormalities.Autoantibodies in ASD autism risk	Evidence concludes that optimal levels are important for healthy development, but over-supplementation can lead to negative outcomes [72].
3	Gao Y et al., (2016) [73]	Systematic Review	Evaluation of evidence of FA impact on neurodevelopment	FA supplementation.Maternal red blood cell (RBC) folate levels. Plasma folate	The review suggests a beneficial effect of folic acid supplementation in pregnancy on children’s neurodevelopment [73].
4	Guo B-Q et al., (2019) [74]	Systematic Review and Meta-Analysis	Elucidate the association of maternal FA intake during the prenatal period and ASD risk in offspring	FA intake.Period of FA intake. FA intake and risk of ASDs subtypes. FA supplementation (excluding diet consumption) and risk of ASDs. Geographical area and risk of ASDs	Findings do not support the link between FA supplementation during prenatal period and ASD reduced risk in offspring. In addition, more investigation is needed because of many study limitations [74].
5	Pu D et al., (2013) [75]	Meta-Analysis	Investigation of the MTHFR polymorphisms (C677T and A1298C) and the ASD risk	Meta-Analysis of MTHFR Polymorphisms between ASD children and controls. Distributions of MTHFR C677T/A1298C genotypes. Meta-Analysis of MTHFR C677T/A1298C polymorphisms on risk of ASD patient population based on whether they were from a country with food fortification of FA or not	This meta-analysis found that periconceptional FA supplementation may reduce ASD risk in those with MTHFR 677C>T polymorphisms where an increased risk of ASDs was indicated. The C677T polymorphism was found to be associated with ASDSs only in children from countries without food fortification [75].
6	Cierna AV et al., (2016) [16]	Review	Investigating the methylation of cytosine bases as one of the most stable and crucial forms of epigenetic regulation of the genome	DNA methylation at different regulatory genomic elements across tissues and cell types and during different developmental stages	In genetically susceptible individuals with altered DNA-methylation patterns, a potential protective effect of supplementation taken before conception was suggested [16].
7	Modabbernia A et al., (2017) [8]	Review	Investigating environmental risk factors for ASDs.	Advanced parental age. Pregnancy-related complications and conditions. Environmental risk factors for ASDs, Genetic and epigenetic-related effects	Studies of environmental risk factors were inconclusive as a result of significant methodological limitations [8].
8	Dias CM et al., (2020) [2]	Review	Elucidate how genetic risk affects cellular functioning and clinical phenotypes	Roles of de novo copy number variants and single-nucleotide variants—causing loss-of-function or missense changes. Mosaic single-nucleotide variants. Inherited variants (including common variants). Rare recessive inherited variants. Noncoding variants, both inherited and de novo	Findings underline the need of whole-exome sequencing and further genome studies with increased sample size for better understanding of neuro-developmental disorders [2].
9	Castro K et al., (2016) [76]	Review	Evaluation of serum nutrient levels and nutritional interventions targeting ASDs.	Folate intake.Serum homocysteine and folate levels.Oral folinic acid supplementation.Urine homocysteine level in children	Inconsistent conclusions were found regarding the association of FA supplementation during pregnancy and ASDs [76].
10	Waye MMY et al., (2017) [6]	Review	Summary of genetic and epigenetic ASDs studies	Environmental risk factors. Genetic risk factors	Study evaluation concluded that although Fragile X, SHANK3, CASPR2 has been linked to ASDs risk, also folate based dietary intervention and environmental pollutants reduction might help to suppress epigenetic changes during maternity. Further study of autoantibodies against Caspr2 and folate receptor alpha has been proposed as important therapeutic targets [6].
11	Schaevitz LR et al., (2012) [77]	Review	Focus on DNA methylation	Genetic polymorphisms. Levels of nutrients in parents and children with ASDs	Evidence underlined the important role of both nutrition and genetic components of the C_1_ metabolic pathway on increasing susceptibility to ASDs. Further studies are needed to better understand the different risk factors and the critical periods most essential for normal development of the brain [77].
12	Paul L et al., (2017) [78]	Review	Summarize interaction between folate and vitamin B_12_ on health consequences	Folate status and biochemical markers of vitamin B_12_ insufficiency. Clinical outcomes associated with vitamin B_12_ deficiency, B vitamin imbalance during pregnancy	Negative health consequences, especially in women during pregnancy and their offspring, have been associated with impaired folate status or intake and vitamin B_12_ status or intake [78].
13	Neggers Y et al., (2014) [79]	Review	Investigation of FA and autism risk	Frequency of MTHFR alleles 677C→*T*1298A→*C* in cases and controls. Plasma levels of folate metabolites. Serum folate, cerebrospinal folate, CSF 5MTHF folate receptor (FR) autoantibodies. Consumption of prenatal multivitamins and nutrients from 3 months before conception during pregnancy. Maternal FAintake. Serum FR blocking. Autoimmune antibody levels	Although results show a positive link of perinatal FA supplementation in reducing ASD incidence, recent studies underline the importance of further studies to understand the modulative role of high maternal FA intake in DNA methylation in ASD and ASD-related traits [79].
14	Chaste P et al., (2012) [7]	Review	Summary of genetic, epigenetic, and environmental risk factors related to autism	Genetic risk factors. Environmental risk factors. Gene-environment interaction	Conclusions are inconsistent as further studies are needed to better characterize the impact of environmental factors, although an additive or multiplicative effect has been indicated [7].
15	Frye RE et al., (2017) [80]	Review	Investigation on biomarkers used to detect folate abnormalities	Polymorphisms in folate genes related to autism environment—genome interaction. Folate metabolism FRAAs in pregnancy. Folate and ASDs	Evidence underlined the need of further studies for specific biomarkers of the folate pathway that might help to detect ASDs early and diagnose ASDs, as the abnormality of FA metabolism has a potential impact in ASD offspring. Thus, specific type and dose of folate and other cofactors could be used for treating or preventing ASD traits [80].

## Data Availability

No new data were created in this study. Data sharing is not applicable to this article.
